# Impact of Meconium-Stained Amniotic Fluid on Neonatal Outcome in a Tertiary Hospital

**DOI:** 10.7759/cureus.24464

**Published:** 2022-04-25

**Authors:** Sadia Parween, Dipali Prasad, Poonam Poonam, Rizwan Ahmar, Archana Sinha, Ranjana Ranjana

**Affiliations:** 1 Obstetrics and Gynecology, Indira Gandhi Institute of Medical Sciences, Patna, IND; 2 Pediatric Medicine, Indira Gandhi Institute of Medical Sciences, Patna, IND

**Keywords:** nicu admissions, apgar score, high-risk pregnancy, meconium aspiration syndrome, meconium stained amniotic fluid

## Abstract

Objective

The aim of this study was to determine the perinatal outcome of pregnant patients complicated with meconium-stained amniotic fluid (MSAF) compared with clear amniotic fluid.

Methodology

This prospective cross-sectional study was conducted in the Department of Obstetrics and Gynecology in collaboration with the Department of Pediatrics at Indira Gandhi Institute of Medical Sciences, Patna, India, from September 2016 to January 2018. A total of 200 patients were included in the study after taking their written consent. Out of these 200 patients, 100 patients had MSAF, and the other 100 patients with clear liquor were taken as controls after fulfilling the inclusion and exclusion criteria. These two groups of patients were compared regarding various maternal and neonatal parameters. These parameters were compared and tested statistically for significance.

Results

Among the 100 patients with MSAF, 20 patients had grade 1 meconium (X), 22 patients had grade 2 meconium (Y), and 58 patients had grade 3 meconium (Z). The majority of patients in the MSAF group were primigravida and more than 25 years of age. In addition, 47% of patients in the MSAF group had some associated high-risk factors and 50% of patients had non-reassuring fetal heart rate patterns, and among these, 39 patients had grade 3 MSAF (X). In the MSAF group, 49% of patients had undergone lower segment cesarean section (LSCS), whereas in the non-MSAF group, it was 37%. Also, 30% of babies in the MSAF group and 13% in the non-MSAF group had neonatal intensive care unit (NICU) admission; 22% of babies in the MSAF group and 12% of babies in the non-MSAF group had an adverse neonatal outcome. Meconium aspiration syndrome was present in 14% of the patients in the MSAF group, and among these, two babies had neonatal death and both had severe birth asphyxia. In the non-MSAF group, there was one neonatal death due to neonatal sepsis. However, after statistically analyzing the neonatal outcome in both the groups, there was no statistical difference between the two groups (p<0.001).

Conclusion

MSAF is associated with increased frequency of operative delivery, poor neonatal outcomes, and increased NICU admission. Management of labor with MSAF requires appropriate intrapartum care with continuous fetal heart rate monitoring, and this can reduce unnecessary cesarean sections in patients with MSAF.

## Introduction

Amniotic fluid is a fluid that surrounds the baby in the uterus, thus providing it a protective and low resistance environment. Amniotic fluid is secreted from fetal skin, amniotic membranes, and fetal urine. Meconium is a dark green liquid passed normally by the newborn baby and contains bile, mucus, and epithelial cells. When the fetus is under some stress, meconium is passed into the amniotic fluid. Its presence is a sign of fetal compromise and is associated with increased perinatal morbidity and mortality [1]. Meconium-stained amniotic fluid (MSAF) is associated with a higher rate of instrumental delivery, cesarean delivery, low birth weight, fetal distress, neonatal intensive care unit (NICU) admission rate, and neonatal death [2].

MSAF usually complicates 13% to 16% of deliveries [3]. Meconium aspiration syndrome (MAS) occurs when the baby aspirates the meconium and it is present in approximately 2 to 10% of all cases of MSAF [4]. Neonatal death occurs in around 12% of infants with MAS [5]. The presence of MSAF may represent the normal maturation of the gastrointestinal tract. It may also be present in conditions of fetal distress due to an acute or chronic hypoxic event [4,6].

When the fetus has an acute or chronic hypoxic event, there is a passage of meconium from the fetus into the amniotic fluid. Many factors such as maternal hypertension, pre-eclampsia, placental insufficiency, oligohydramnios, postdated pregnancy, and maternal drug abuse result in the passage of MSAF [7].

A well-designed study is required as it is evident that MSAF is associated with poor perinatal outcomes. Still, there is much confusion regarding the management of labor associated with MSAF, thus leading to unnecessary cesarean sections. Though some studies have been conducted on the subject matter in many parts of India, very few well-designed comparative studies have been conducted in the northeastern region of India. One such study was conducted by Mundhra and Agarwal in Shillong [3]. The present study aimed to determine the impact of MSAF perinatal outcomes and maternal outcomes and compare it with that of clear amniotic fluid at a tertiary referral hospital in Patna, Bihar, India.

The primary objective of the study was to determine the perinatal outcome of pregnant patients complicated with MSAF compared with clear amniotic fluid. The secondary objective was to determine the risk factors during pregnancy and mode of delivery of laboring mothers with MSAF compared with clear amniotic fluid.

## Materials and methods

Study design and participants

This prospective cross-sectional study was conducted in the Department of Obstetrics and Gynecology in collaboration with the Department of Pediatrics at the Indira Gandhi Institute of Medical Sciences, Patna, Bihar, India, from September 2016 to January 2018. The study was started after getting ethical clearance from the institutional ethical committee (IEC letter no 535/Acad, dated 20.06.2016).

A total of 200 patients were included in the study after taking their written consent. Out of these 200 patients, 100 patients had MSAF, while the other 100 patients with clear liquor were taken as controls.

Inclusion and exclusion criteria

Patients were included in the study if they had a singleton pregnancy, had a cephalic presentation, and were above 34 weeks of gestation with the presence of MSAF during labor. A matched group of subjects with clear amniotic fluid were also included. Exclusion criteria for this study included pregnancy with congenital fetal abnormalities, stillbirth, breech presentation, and gestation age <34 weeks.

Study procedure

All demographic details of the patients were recorded. Patients were examined thoroughly, and the findings were noted down in a proforma. Regular fetal heart rate (FHR) monitoring was conducted. After considering all the obstetrical conditions, the mode of delivery of the patients in both groups was decided and noted down. Maternal risk factors were noted down. Women were classified into two groups: one group with MSAF, and the other group with clear amniotic fluid. MSAF was further divided into three grades: grade I was thin yellow color meconium with no particulate matter, grade II was light green color with few particulate matters, and grade III was thick paste-like dark green-colored meconium with excess particulate matter. The condition of neonates, i.e., birth weight, Apgar score, general condition, need for admission in neonatal ICU, and neonatal complications and outcome were also recorded. These parameters were compared between the two groups and tested statistically for significance.

Statistical analysis

Data were coded and recorded on an MS Excel spreadsheet. For data analysis, we used SPSS Version 23 (IBM Corp., Armonk, NY). For descriptive statistics, we used means/standard deviations or medians/interquartile ranges for continuous variables, while we used frequencies and percentages for categorical variables. Data were presented graphically wherever appropriate for data visualization using histograms, box-and-whiskers plots, and column charts for continuous data, and bar charts and pie charts for categorical data. Group comparisons for continuously distributed data were made using the independent sample t-test when comparing two groups. If data were found to be non-normally distributed, appropriate non-parametric tests in the form of the Wilcoxon test were used. The chi-square test was used for group comparisons of categorical data. In case the expected frequency in the contingency tables was found to be <5 for >25% of the cells, Fischer's exact test was used instead. Linear correlation between two continuous variables was explored using Pearson's correlation (if the data were normally distributed) and Spearman's correlation (for non-normally distributed data). Statistical significance was kept at p < 0.05.

## Results

Among the 100 patients with MSAF, 20 patients had grade 1 meconium (X), 22 patients had grade 2 meconium (Y), and 58 patients had grade 3 meconium (Z),

Table 1 shows that the variable age (years) was not normally distributed in the two subgroups of the variable MSAF. Thus, non-parametric tests (Wilcoxon-Mann-Whitney U test) were used to make group comparisons. The mean (SD) of age (years) in the MSAF group was 24.74 (4.46) and that in the non-MSAF group was 26.14 (3.88). The median (IQR) of age (years) in the MSAF group was 24 (22-28) and that in the non-MSAF group was 26 (24-29). The age (years) in the MSAF group ranged from 18 to 40 and that in the non-MSAF ranged from 19 to 37. There was a significant difference between the two groups in terms of age (years) (W = 3,858.500, p = 0.005), with the median age (years) being highest in the non-MSAF group. Strength of association (point-biserial correlation) = 0.17 (small effect size).

**Table 1:** Comparison of the two subgroups of the variable MSAF in terms of age (years) (n = 200) MSAF, meconium-stained amniotic fluid; SD, standard deviation; IQR, interquartile range

Age (Years)	MSAF		Wilcoxon-Mann-Whitney U Test	
	Present	Absent	W	p-Value
Mean (SD)	24.74 (4.46)	26.14 (3.88)	3,858.50	0.005
Median (IQR)	24 (22-28)	26 (24-29)		
Range	18-40	19-37		

The chi-square test was used to explore the association between MSAF and parity (Table 2). There was a significant difference between the various groups in terms of distribution of parity (χ^2^ = 6.152; p = 0.046). Strength of association between the two variables (Cramer's V) = 0.18 (low association). Strength of association between the two variables (bias-corrected Cramer's V) = 0.14 (low association). In the MSAF group, 64.0% of the participants were primigravida and 36% of the participants were multigravida. In the non-MSAF group, 54.0% of the participants were primigravida and 46% of the participants were multigravida. Participants in the MSAF group had a larger proportion of primigravida patients as compared to the other group.

**Table 2:** Association between MSAF and parity (n = 200) MSAF, meconium-stained amniotic fluid; G1, gravida 1; G2, gravida 2; G3, gravida 3

Parity	MSAF			Chi-Square Test	
	Present	Absent	Total	χ^2^	p-Value
G1	64 (64.0%)	54 (54.0%)	118 (59.0%)	6.152	0.046
G2	26 (26.0%)	23 (23.0%)	49 (24.5%)		
≥G3	10 (10.0%)	23 (23.0%)	33 (16.5%)		
Total	100 (100.0%)	100 (100.0%)	200 (100.0%)		

The chi-square test was used to explore the association between MSAF and high-risk factors (Table 3). There was a significant difference between the groups in terms of distribution of high-risk factors: any (χ^2^ = 8.580; p = 0.003). Strength of association between the two variables (Cramer's V) = 0.21 (low association). Strength of association between the two variables (bias-corrected Cramer's V) = 0.2 (low association). Participants in the MSAF group had a larger proportion of high-risk factors as compared to the participants in the non-MSAF group. In our study out of 100 patients with MSAF, high-risk factors were present in 47 patients. Out of these 47 patients, 14 patients had postdated pregnancy, 18 had premature rupture of membrane (PROM), 6 had oligohydramnios/anhydramnios, 5 patients each had preeclampsia and preterm labor, 3 had anemia in pregnancy, 2 patients were Rh-negative, 1 patient had gestational diabetes mellitus (GDM), and 8 patients had other miscellaneous risk factors. Among the other 100 patients with clear liquor, 27 patients (27%) had associated high-risk factors. Out of these 27 patients, 6 patients had postdated pregnancy, 5 patients had oligohydramnios, 3 patients had PROM, 4 patients had preterm labor, 3 patients had preeclampsia, 1 patient each had GDM and anemia, 3 patients had Rh-negative pregnancy, and 2 patients each had cardiac disease, antepartum hemorrhage, and other miscellaneous risk factors.

**Table 3:** Association between MSAF and high-risk factors MSAF, meconium-stained amniotic fluid; PROM, premature rupture of membrane; RHD, rheumatic heart disease

High-Risk Factors: Any	MSAF		p-Value
	Present	Absent	
Overall	47 (47.0%)	27 (27.0%)	0.003
Postdated pregnancy	14 (14.0%)	6 (6.0%)	0.059
Oligohydramnios/anhydramnios	6 (6.0%)	5 (5.0%)	0.756
PROM	18 (18.0%)	3 (3.0%)	0.001
Late preterm	5 (5.0%)	4 (4.0%)	1
Preeclampsia/eclampsia	5 (5.0%)	3 (3.0%)	0.721
Gestational diabetes mellitus	1 (1.0%)	1 (1.0%)	1
Anemia in pregnancy	3 (3.0%)	1 (1.0%)	0.621
RH-negative pregnancy	2 (2.0%)	3 (3.0%)	1
Cardiac disease (RHD)	0 (0.0%)	2 (2.0%)	0.497
Antepartum hemorrhage	0 (0.0%)	2 (2.0%)	0.497
Others	8 (8.0%)	2 (2.0%)	0.052

We used the chi-square test to explore the association between MSAF and FHR pattern (Table 4). There was a significant difference between the various groups in terms of the distribution of FHR patterns (χ^2^ = 33.754, p < 0.001). Strength of association between the two variables (Cramer's V) = 0.41 (moderate association). Strength of association between the two variables (bias-corrected Cramer's V) = 0.41 (moderate association). In the MSAF group, 50.0% of the participants had a reassuring FHR pattern and 50.0% had a non-reassuring FHR pattern. In the non-MSAF group, 88.0% of the participants had a reassuring FHR pattern and only 12.0% had a non-reassuring FHR pattern. Participants in the non-MSAF group had a larger proportion of reassuring FHR patterns, and participants in the MSAF group had a larger proportion of non-reassuring FHR patterns.

**Table 4:** Association between MSAF and FHR pattern (n = 200) MSAF, meconium-stained amniotic fluid; FHR, fetal heart rate

FHR Pattern	MSAF			Chi-Square Test	
	Present	Absent	Total	χ2	p-Value
Reassuring	50 (50.0%)	88 (88.0%)	138 (69.0%)	33.754	<0.001
Non-Reassuring	50 (50.0%)	12 (12.0%)	62 (31.0%)		
Total	100 (100.0%)	100 (100.0%)	200 (100.0%)		

The chi-square test was used to explore the association between grade of meconium and FHR pattern (Table 5). There was a significant difference between the various groups in terms of the distribution of FHR patterns (χ^2^ = 18.333; p < 0.001). Out of 100 participants in the MSAF group, 85.0% in the X group had reassuring FHR patterns and 15.0% had non-reassuring FHR patterns. In the Y group, 63.6% of the participants had a reassuring FHR pattern and 36.4% had a non-reassuring FHR pattern. In the Z group, 32.8% of the participants had a reassuring FHR pattern and 67.2% had a non-reassuring FHR pattern. Participants in the X group had the largest proportion of reassuring FHR patterns, whereas participants in the Z group had the largest proportion of non-reassuring FHR patterns.

**Table 5:** Association between grade of meconium and FHR pattern (n = 100) FHR, fetal heart rate

FHR Pattern	Grade of Meconium				Chi-Square Test	
	X Group	Y Group	Z Group	Total	χ2	p-Value
Reassuring	17 (85.0%)	14 (63.6%)	19 (32.8%)	50 (50.0%)	18.333	<0.001
Non-reassuring	3 (15.0%)	8 (36.4%)	39 (67.2%)	50 (50.0%)		
Total	20 (100.0%)	22 (100.0%)	58 (100.0%)	100 (100.0%)		

Fisher's exact test was used to explore the association between MSAF and mode of delivery as more than 20% of the total number of cells had an expected count of less than 5 (Table 6). There was no significant difference between the various groups in terms of distribution of mode of delivery (χ^2^ = 4.156; p = 0.122).Strength of association between the two variables (Cramer's V) = 0.14 (low association). Strength of association between the two variables (bias-corrected Cramer's V) = 0.1 (little/no association). In the MSAF group, 47.0% of the participants had a normal vaginal delivery (NVD), 49.0% had undergone LSCS, and 4.0% had operative vaginal delivery. In the non-MSAF group, 61.0% of the participants had NVD, 37.0% had undergone LSCS, and 2.0% had operative vaginal delivery.

**Table 6:** Association between MSAF and mode of delivery (n = 200) MSAF, meconium-stained amniotic fluid; NVD, normal vaginal delivery; LSCS, lower segment cesarean section; VD, vaginal delivery

Mode of Delivery	MSAF			Fisher's Exact Test	
	Present	Absent	Total	χ^2^	p-Value
NVD	47 (47.0%)	61 (61.0%)	108 (54.0%)	4.156	0.122
LSCS	49 (49.0%)	37 (37.0%)	86 (43.0%)		
Operative VD	4 (4.0%)	2 (2.0%)	6 (3.0%)		

The variable Apgar score (1 minute) was not normally distributed in the two subgroups of the variable MSAF. Thus, non-parametric tests (Wilcoxon-Mann-Whitney U test) were used to make group comparisons (Table 7). The mean (SD) of the APGAR score (1 minute) in the MSAF group was 7.20 (0.92) and that in the non-MSAF group was 7.53 (1.03). The median (IQR) Apgar score (1 minute) in the MSAF group was 7 (7-8) and that in the non-MSAF group was 8 (7-8). The Apgar score (1 minute) in the MSAF group ranged from 4 to 9 and that in the non-MSAF group ranged from 5 to 9. There was a significant difference between the two groups in terms of Apgar score (1 minute) (W = 6,060.000; p = 0.006), with the median APGAR score (1 minute) being highest in the non-MSAF group. Strength of association (point-biserial correlation) = 0.17 (small effect size).

**Table 7:** Comparison of the two subgroups of the variable MSAF in terms of Apgar score (1 minute) (n = 200) MSAF, meconium-stained amniotic fluid; SD, standard deviation; IQR, interquartile range

Apgar Score (1 Minute)	MSAF		Wilcoxon-Mann-Whitney U Test	
	Present	Absent	W	p-Value
Mean (SD)	7.20 (0.92)	7.53 (1.03)	6060	0.006
Median (IQR)	7 (7-8)	8 (7-8)		
Range	4 – 9	5 – 9		

The variable Apgar score (5 minutes) was not normally distributed in the two subgroups of the variable MSAF. Thus, non-parametric tests (Wilcoxon-Mann-Whitney U test) were used to make group comparisons (Table 8). The mean (SD) of Apgar score (5 minutes) in the MSAF group was 8.58 (0.84) and that in the non-MSAF group was 8.77 (0.85). The median (IQR) of Apgar score (5 minutes) in the MSAF group was 9 (8-9) and that in the non-MSAF group was 9 (8-9). The Apgar score (5 minutes) in the MSAF group ranged from 5 to 10 and that in the non-MSAF group ranged from 6 to 10. There was no significant difference between the groups in terms of Apgar score (5 minutes) (W = 5,562.500; p = 0.106). Strength of association (point-biserial correlation) = 0.11 (small effect size).

**Table 8:** Comparison of the two subgroups of the variable MSAF in terms of Apgar score (5 minutes) (n = 200) MSAF, meconium-stained amniotic fluid; SD, standard deviation; IQR, interquartile range

APGAR Score (5 Minutes)	MSAF		Wilcoxon-Mann-Whitney U Test	
	Present	Absent	W	p-Value
Mean (SD)	8.58 (0.84)	8.77 (0.85)	5562.5	0.106
Median (IQR)	9 (8-9)	9 (8-9)		
Range	05-Oct	06-Oct		

The chi-square test was used to explore the association between MSAF and NICU admission (Table 9). There was a significant difference between the various groups in terms of distribution of NICU admission (χ^2^ = 8.562; p = 0.003). Strength of association between the two variables (Cramer's V) = 0.21 (low association). Strength of association between the two variables (bias-corrected Cramer's V) = 0.19 (low association). Babies of 30.0% of the participants in the MSAF group had NICU admission, whereas babies of 13.0% of the participants in the non-MSAF had NICU admission. Participants in the MSAF group had a larger proportion of NICU admissions.

**Table 9:** Association between MSAF and NICU admission (n = 200) MSAF, meconium-stained amniotic fluid; NICU, neonatal intensive care unit

NICU Admission	MSAF			Chi-Square Test	
	Present	Absent	Total	χ^2^	p-Value
Yes	30 (30.0%)	13 (13.0%)	43 (21.5%)	8.562	0.003
No	70 (70.0%)	87 (87.0%)	157 (78.5%)		
Total	100 (100.0%)	100 (100.0%)	200 (100.0%)		

The chi-square test was used to explore the association between grade of meconium and NICU admission (Table 10). There was a significant difference between the various groups in terms of distribution of NICU admission (χ^2^ = 6.938; p = 0.031). Strength of association between the two variables (Cramer's V) = 0.26 (low association). Strength of association between the two variables (bias-corrected Cramer's V) = 0.22 (low association). Participants in the Z group had the largest proportion of NICU admission (23), whereas participants in the X group had the least proportion of NICU admission (2).

**Table 10:** Association between grade of meconium and NICU admission (n = 100) NICU, neonatal intensive care unit

NICU Admission	Grade of Meconium				Chi-Square Test	
	X Group	Y Group	Z Group	Total	χ2	p-Value
Yes	2 (10.0%)	5 (22.7%)	23 (39.7%)	30 (30.0%)	6.938	0.031
No	18 (90.0%)	17 (77.3%)	35 (60.3%)	70 (70.0%)		
Total	20 (100.0%)	22 (100.0%)	58 (100.0%)	100 (100.0%)		

Fisher's exact test was used to explore the association between MSAF and neonatal outcome as more than 20% of the total number of cells had an expected count of less than 5 (Table 11). There was a significant difference between the various groups in terms of distribution of neonatal outcomes (χ^2^ = 24.436; p < 0.001). Strength of association between the two variables (Cramer's V) = 0.35 (moderate association). Strength of association between the two variables (bias-corrected Cramer's V) = 0.27 (low association). In the MSAF group, 14 (14%) of the neonates developed MAS. Out of these 14 neonates with MAS, two babies died. One baby in the MSAF group had respiratory distress syndrome (RDS), two had neonatal sepsis, two had neonatal hypoglycemia, one had transient tachypnea of newborn (TTN), and two had neonatal jaundice. Also, 78 neonates had an uneventful outcome. In the non-MSAF group, seven neonates had RDS, two had neonatal sepsis, one had neonatal hypoglycemia, and two had TTN. Among the two babies who had neonatal sepsis, one baby died. Also, 88 neonates had an uneventful outcome.

**Table 11:** Association between MSAF and neonatal outcome (n = 200) MSAF, meconium-stained amniotic fluid; MAS, meconium aspiration syndrome; RDS, respiratory distress syndrome; TTN, transient tachypnea of the newborn; DCT, direct Coomb's test

Neonatal Outcome	MSAF			Fisher's Exact Test	
	Present	Absent	Total	χ2	p-Value
Uneventful	78 (78.0%)	88 (88.0%)	166 (83.0%)	24.436	<0.001
MAS	14 (14.0%)	0 (0.0%)	14 (7%)		
RDS	1 (1.0%)	7 (7.0%)	8 (4.0%)		
Neonatal sepsis	2 (2.0%)	2 (2.0%)	4 (2.0%)		
Neonatal hypoglycemia	2 (2.0%)	1 (1.0%)	3 (1.5%)		
hypoglycemia with TTN	1 (1.0%)	0 (0.0%)	1 (0.5%)		
Neonatal jaundice	1 (1.0%)	0 (0.0%)	1 (0.5%)		
Rh incompatibility, jaundice, DCT +	1 (1.0%)	0 (0.0%)	1 (0.5%)		
TTN	0 (0.0%)	2 (1.0%)	2 (1)		
Total	100 (100.0%)	100 (100.0%)	200 (100.0%)		

## Discussion

In the present study, various parameters were assessed to find out the effect of MSAF on the mode of delivery and immediate neonatal outcome. Various high-risk factors associated with MSAF were also assessed. A significant association between MSAF and abnormal FHR patterns, increased rate of cesarean section, and low Apgar score has been reported in various studies [1-3,7].

In terms of age in the MSAF group, the present study correlates with the study conducted by Rafia et al. as the maximum number of participants with MSAF were younger than 25 years [8]. This was in contrast to the studies conducted by Lee et al. and Addisu et al. in which the maximum number of participants with MSAF were older than 25 years [4,9].

This study had a similar finding to the study conducted by Unnisa et al. and Becker et al. which reported a higher incidence of MSAF in primigravidas [10,11]. This was in contrast to the study conducted by Mundhra and Agarwal, in which a slightly higher incidence of MSAF was seen in multigravidas (51.52%) [3].

In our study, 14% of patients with MSAF had postdated pregnancies. This correlated with the study conducted by Rafia et al. and Singh et al. in which they had 13% and 12% postdated pregnancies, respectively [8,12].

In our study, 50% of the patients with MSAF had fetal distress, and out of these 50 cases, 39 cases had thick meconium-stained liquor. In the clear liquor group, only 12% of patients had fetal distress. This correlated with the study conducted by Rafia et al. in which fetal distress was present in 65.8% of patients with MSAF [8]. This is, in contrast, to a study conducted by Qadir et al., in which fetal distress was present in 29.6% of cases with MSAF [13].

In our study, there were 47 NVD, 49 LSCS, and 4 operative vaginal delivery in the MSAF group, while there were 61 NVD, 37 LSCS, and 2 operative vaginal delivery in the clear liquor group. Although the number of LSCS was more in the MSAF group, the difference was not statistically significant. The reason for a higher rate of cesarean section in the non-MSAF group is that our institute is a referral tertiary care center and more than 50% of our patients have some associated high-risk factors. Out of the 49 LSCS in the MSAF group, 37 patients had grade 3 MSAF. Our study correlates with the study conducted by Qadir et al. and Jain et al. in which the cesarean rates were 46.3% and 44.6%, respectively [13,14]. Other studies such as those Kumar et al. and Wong et al. also reported similar findings [15,16]. Our result was in contrast to the studies conducted by Shaikh et al., in which the cesarean rate was 82% [17]. Although having a higher rate of grade 3 MSAF and a non-reassuring FHR pattern, the cesarean rate was comparatively low in our study.

In our study, 52% of babies were male and 48% were female, with a male:female ratio of 1.08:1 in the MSAF group. This was similar to a study conducted by Afsar et al., in which 57.5% were male babies and 42.5% were female babies [18]. This was in contrast to David et al.'s study, in which 48% were male babies and 52% were female babies [19].

In our study, 30 (30%) of the babies of the MSAF group and 13 (13%) of babies of the non-MSAF group required NICU admission. Out of the 30 babies in the MSAF group requiring NICU admission, 23 (76%) babies had grade 3 MSAF. The result was comparable to studies conducted by Qadir (21.21%) and Odongo et al. (24.5%) [13,20]. Like our study, other studies also showed a higher rate of NICU admission in babies with thick MSAF.

In our study, poor neonatal outcome was present in 22% of patients in the MSAF group and 12% in the non-MSAF group, and there was no statistical difference between the two groups. There was one neonatal death in the MSAF group, which was due to MAS. There was one stillbirth in the non-MSAF group, in which there was fetal distress for which LSCS was performed, and there was no high-risk factor in the patient.

MAS was present in 14% of cases in the MSAF group in our study. A similar finding was present in the study conducted by Qadir et al. (18.8%) [13]. In contrast to our study, the incidence of MAS was lower in the study by Tolu et al. (6.3%) [21], whereas a higher incidence of MAS was reported in the study conducted by Shaikh et al. (46%) [17].

There were two (2%) deaths in the MSAF group, and both had MAS. This result was similar to the study conducted by Jain et al. [14] in which early neonatal death was 0.54% in the MSAF group. In contrast to this, there was 9% early neonatal death in the study by Tolu et al. [21]. Neonatal death in MAS was 14.2% in our study, which was similar to a study conducted by Davis et al., in which it was 12% [5].

In our study, the adverse neonatal outcome was present in 22% patients in the MSAF group and 12% patients in the non-MSAF grouppatients. Out of this 22% in the MSAF group, two had neonatal death due to MAS leading to severe birth asphyxia. Out of this 12% in the non-MSAF group, one had neonatal death due to neonatal sepsis. After statistically analyzing the neonatal outcome in both the groups, there was no statistical difference found between the two groups (p < 0.001).

The reason for the increased rate of LSCS and NICU admission in the non-MSAF group maybe because our institute is a tertiary care referral center with more than 50% of the patients coming to our hospital having some associated high-risk factors.

The relationship between MSAF with poor fetal outcomes and associated risk factors has been already extensively studied. We conducted this study as we wanted to look for the outcomes in our institute and also because very few well-designed comparative studies on the subject matter have been conducted in our region.

Limitations

As our study was a cross-sectional study, there are certain limitations to it, and hence a temporal relationship between MSAF and explanatory variables may not be possible to establish. The results of our study might not be representative of other institutions and the community as it was conducted in a single referral hospital. Another limitation is the small sample size.

## Conclusions

MSAF is associated with increased frequency of operative delivery, poor neonatal outcomes, and increased NICU admission. Identification of the high-risk factors is important, and timely referral of the patients to centers with proper neonatal care facilities with mechanical ventilators reduces neonatal morbidity and mortality. Management of labor with MSAF requires appropriate intrapartum care with continuous FHR monitoring. The mere presence of meconium in amniotic fluid is not an indication of cesarean section. It depends on the grade of meconium, with grade 3 being mostly responsible for non-reassuring FHR pattern. Close monitoring of patients with MSAF reduces unnecessary cesarean section with successful vaginal delivery and thus reduces maternal and neonatal morbidity.
